# You Have One Reminder: Self-Manage Your Pain

**DOI:** 10.1093/geroni/igaa057.664

**Published:** 2020-12-16

**Authors:** Marcia Shade, Kyle Rector, Rasila Soumana, Kevin Kupzyk

**Affiliations:** 1 University of Nebraska Medical Center, Omaha, Nebraska, United States; 2 University of Iowa, Iowa city, Iowa, United States

## Abstract

Pain has a significant impact in the lives of aging adults. As the population of America grows older, they need to be encouraged to use pain self-management strategies. Technology is an option, but current pain management applications have usability limitations in older adults. This feasibility study describes the usability of voice assistant reminders for pain self-management. We enrolled 15 community-dwelling aging adults with chronic pain. Participants created two pre-determined voice assistant reminder tasks: 1) to take scheduled pain medication, and 2) to write in a pain diary. We collected data on demographics, pain, confidence of managing symptoms, and objective ease of use. After four weeks, we collected information about subjective ease of use and usefulness of the voice assistant. Participants were mostly female, average age 65 years; reporting moderate pain severity 4.58 (SD 2.29) and pain interference 3.94 (SD 2.62). The mean PROMIS self-efficacy for managing symptoms score was 50.8 (SD 8.2). Voice assistant usability was above average (78 out of 100). The median time to make a voice assistant profile was five minutes (SD 7.5), with a median of seeking help two times. No significant relationships were found between pain and usability. Three participants made physical activity and distraction reminders to self-manage pain. Voice assistant reminders were perceived as consistent, easy to set up and helpful for accountability. Older users may provide helpful feedback for development and testing of voice assistant software for pain self-management. Voice assistants may provide helpful reminders that encourage the completion of pain self-management strategies.

